# Occurrence and temporal overlap of sympatric jungle cats and leopard cats in Parsa‒Koshi Complex, Nepal

**DOI:** 10.1038/s41598-024-52644-w

**Published:** 2024-01-29

**Authors:** Hari Prasad Sharma, Bishnu Prasad Bhattarai, Sandeep Regmi, Shivish Bhandari, Dipendra Adhikari, Bishnu Aryal, Krishna Tamang, Amrit Nepali, Sabin K. C., Basudha Rawal, Sagar Parajuli, Bashu Dev Baral, Surya Devkota, Sabina Koirala, Jerrold L. Belant, Hem Bahadur Katuwal

**Affiliations:** 1 Central Department of Zoology, Institute of Science and Technology, Tribhuvan University, Kirtipur, Kathmandu, Nepal; 2 Nepal Zoological Society, Kirtipur, Kathmandu, Nepal; 3 Center for Integrative Conservation, Xishuangbanna Tropical Botanical Garden, Chinese Academy of Sciences, Mengla, 666303 Yunnan China; 4 Department of Biology, Morgan State University, Baltimore, MD 21251 USA; 5 Department of Fisheries and Wildlife, Michigan State University, East Lansing, MI 48824 USA

**Keywords:** Ecology, Zoology, Ecology

## Abstract

Co-occurrence and spatial and temporal overlap of sympatric jungle and leopard cats are influenced by habitat preferences, and interspecific competition. Understanding these factors influence is crucial for developing effective conservation strategies. We conducted a camera survey in Parsa‒Koshi Complex (PKC), Nepal during December 2022–March 2023 to investigate factors influencing occupancy and spatial and temporal overlap between jungle cats (*Felis chaus*) and leopard cats (*Prionailurus bengalensis*). The mean detection probability (*t* = 0.664, *p* = 0.507) did not differ between jungle cats (*p* = 0.500 ± 0.289) and leopard cats (*p* = 0.501 ± 0.288); however, occupancy (*t* = 31.008, *p* < 0.001) was greater for jungle cats (*ψ* = 0.247 ± 0.020) than leopard cats (*ψ* = 0.178 ± 0.019). Jungle cats and leopard cats were positively associated with large predators, and jungle cats were positively associated with human presence and negatively associated with canopy cover. We observed high diel overlap between leopard cats and jungle cats (*Dhat1* = 0.802, norm0CI: 0.720–0.884), with both species largely nocturnal. Co-existence of jungle cats and leopard cats in PKC appears to be facilitated by spatial segregation. These findings provide valuable insights into the complex ecological dynamics and interactions between sympatric jungle and leopard cats.

## Introduction

Co-occurrence of carnivore species present fascinating dynamics and interactions within ecosystems^1^. Carnivore species play a pivotal role in shaping ecosystems and associated processes^2,3^. There are many drivers of species coexistence including resource partitioning^4,5^, competition^6^, and niche differentiation^7,8^. Sympatric carnivores often display adaptations and behaviors^9^, that allow them to use different food sources^10^ and occupy distinct ecological niches^11^ to reduce direct competition^10,12^ and facilitate their cohabitation^13^. In African savannas, sympatric carnivore species such as lions (*Panthera leo*), leopards (*P. pardus*), and African wild dogs (*Lycaon pictus*) share the same habitat^14^. As top predators, carnivores can exert control over prey populations by influencing their distribution and abundance^15^ and regulate trophic cascades^16,17^. However, little is known for meso-carnivores such as jungle cats (*Felis chaus*) and leopard cats (*Prionailurus bengalensis*) that are sympatric across wide portions of their geographic ranges^18,19^.

Sympatric jungle cats and leopard cats, along with other small- to medium-sized carnivores, often exhibit niche differentiation^20^ and adapt their hunting strategies to target different prey^21^. Both cat species occur below 4500 m elevation above sea level, and in Nepal, jungle cats and leopard cats are widely distributed below 4000 and 3254 masl, respectively^18–20^. Where jungle cats and leopard cats co-occur^21,22^, jungle cats prefer open forest and shrubland habitats^23^ and primarily hunt small mammals, birds, and reptiles^24^. In contrast, leopard cats are more adaptable and use habitats such as continuous evergreen, evergreen mosaic, and open dry deciduous forests^25^, targeting similar prey of jungle cat but potentially using different microhabitats^26^. The sympatry of these cat species underscores the importance of resource partitioning and highlights the complexity of predator interactions within ecosystems.

The presence of larger predators can have a notable influence on jungle cats and leopard cats^27,28^. Small carnivores generally exhibit spatial or temporal avoidance to reduce encounters with larger carnivores^29^. Temporal habitat segregation has also been identified as a mechanism to allow co-occurrence of diverse predator guilds^30,31^. Larger predator species can potentially create a competitive dynamic with jungle cats and leopard cats exhibiting spatial and temporal avoidance^32,33^. In addition, the coexistence of humans with these cat species can reduce human-rodent conflicts by their hunting of prey in human-occupied areas^34^. However, loss and fragmentation of natural habitats from human activities can restrict their movements, disrupt foraging patterns, and limit access to prey. Human activities also result in potential threats to jungle cats and leopard cats including poaching and trapping^35^ and collisions with vehicles^36–38^ which can directly impact populations.

We aimed to assess the occupancy patterns of sympatric jungle and leopard cats and identify the drivers shaping it in Parsa‒Koshi Complex (PKC), Nepal, to provide baseline information for informing conservation strategies including habitat management and protected area planning. The PKC includes protected and non-protected areas providing greater opportunities to understand their occupancy in different management regimes. Further we investigated the difference in temporal activity between these sympatric species in PKC.

## Results

We obtained 10,352 images including 3969 of wild mammals. We obtained 81 jungle cat detections at 51 sites and 68 leopard detections at 44 sites; both species were detected at 19 sites. The mean probability of large carnivore detected together was 0.357 ± 0.481(SD), mean number of human detections was 63.1 ± 237.9 (SD), and mean number of livestock detections 36.46 ± 102.17 (SD). Mean canopy cover was 41.6 ± 21.5% across camera locations whereas the mean distance to water body was 2196 ± 2222 m, mean distance to major road was 795 ± 1252 m, and mean distance to nearest human settlement was 3211 ± 1932 m.

### Occupancy analysis

We observed a similar mean detection probability (*t* = 0.664, *p* = 0.507) between jungle cats (*p* = 0.500 ± 0.289; Fig. S2) and leopard cats (*p* = 0.501 ± 0.288), but higher occupancy (*t* = 31.008, *p* < 0.001) for jungle cats (*ψ* = 0.247 ± 0.020) than leopard cats (*ψ* = 0.178 ± 0.019).

**Figure 1:**
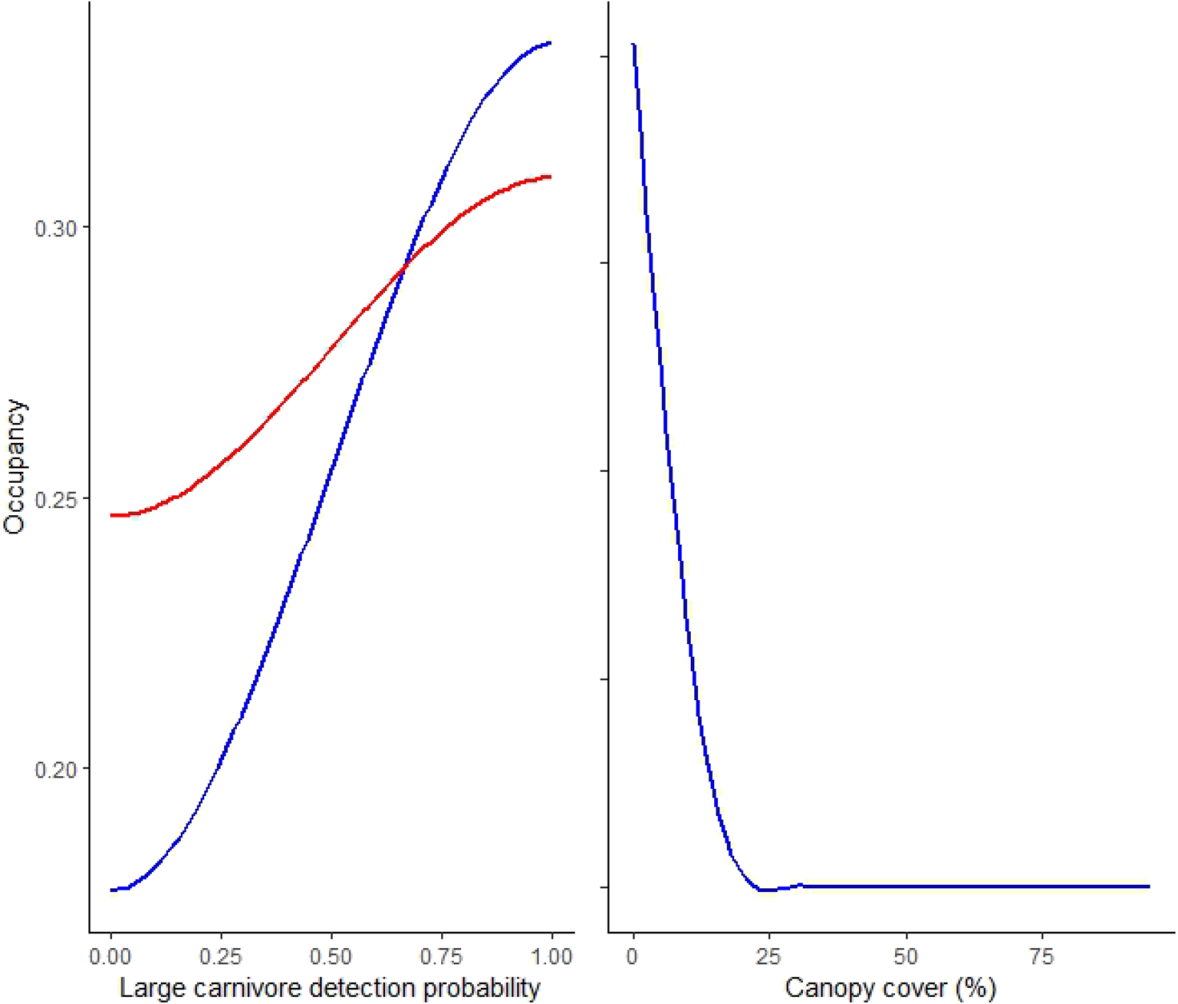
Covariate effects on leopard cat (red lines) and jungle cat (blue lines) occupancy, Parsa–Koshi Complex, Nepal, 2022–2023.

**Figure 2:**
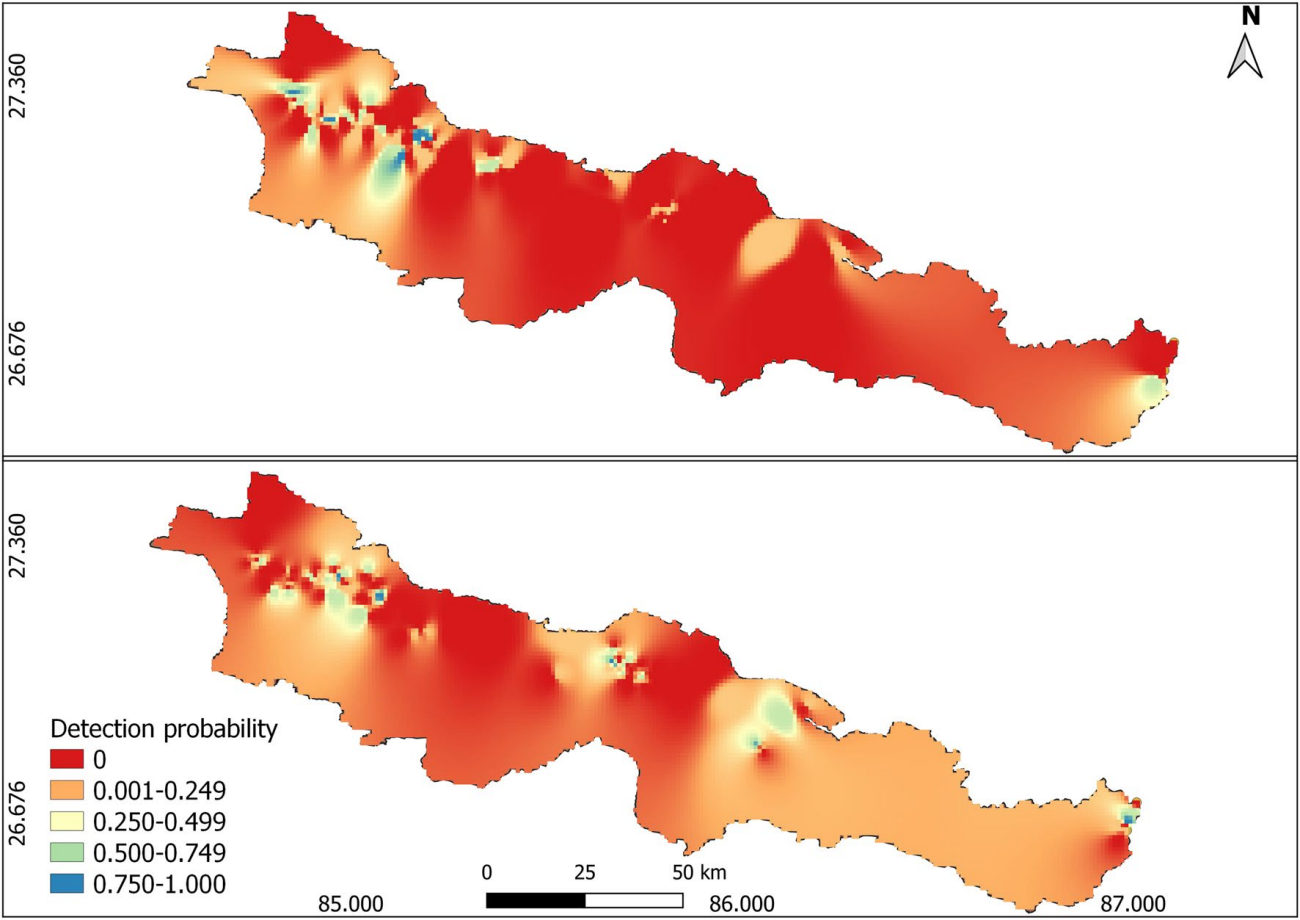
Inverse Distance Weighting (IDW) interpolation based spatial detection probability map of leopard cats (top panel) and jungle cats (bottom panel), Parsa–Koshi Complex, Nepal, 2022–2023. Map created in QGIS 3.24.1 of QGIS Development Team (https://qgis.org/).

Probability of occupancy increased for jungle cats and leopard cats with increasing detections of large predators (Table 1, Fig. 1). Jungle cat occupancy decreased with increasing canopy cover (*βcanopycover* = -0.731 ± 0.341). No other factors influenced occupancy of jungle cats or leopard cats. We observed overall modest but greater detection probability of jungle cats and leopard cats in eastern PKC, with few areas of PKC having high (> 0.50) detection probability for either species (Fig. 2).

**Table 1:** Covariate effects on leopard cat and jungle cat occupancy, Parsa–Koshi Complex, Nepal, 2022–2023.

Model Component	Parameters	Mean	SD	LCI	UCI	Rhat	ESS	overlap0	F
	Leopard cat								
Space Use	Intercept	− 1.534	0.132	− 1.802	− 1.284	1.000	8384	0	1
	Canopy cover	0.103	0.136	− 0.161	0.370	1.000	26,249	1	0.776
	Large predators	0.842	0.124	0.602	1.091	1.000	6956	0	1.000
	Livestock	0.223	0.120	− 0.020	0.454	1.000	7806	1	0.964
	Human	− 0.285	0.221	− 0.813	0.044	1.000	42,000	1	0.943
	Jungle cat	0.122	0.114	− 0.104	0.345	1.000	42,000	1	0.859
Detection	Intercept	0.002	1.003	− 1.964	1.966	1.000	42,000	1	0.504
	Distance to water	− 0.014	2.889	− 4.751	4.750	1.000	21,795	1	0.503
	Distance to road	0.000	2.891	− 4.742	4.747	1.000	42,000	1	0.499
	Distance to settlement	0.008	2.883	− 4.742	4.741	1.000	16,741	1	0.503
	Jungle cat								
Space Use	Intercept	− 1.117	0.107	− 1.332	− 0.910	1.000	18,026	0	1
	Canopy cover	− 0.307	0.112	− 0.529	− 0.088	1.000	12,333	0	0.997
	Large predators	0.313	0.113	0.093	0.535	1.000	42,000	0	0.997
	Livestock	− 0.067	0.119	− 0.313	0.154	1.000	9851	1	0.705
	Human	0.032	0.109	− 0.190	0.244	1.000	13,412	1	0.629
	Leopard cat	0.078	0.108	− 0.138	0.290	1.000	7917	1	0.766
Detection	Intercept	0.006	1.000	− 1.955	1.975	1.000	20,792	1	0.502
	Distance to water	0.010	2.888	− 4.748	4.751	1.000	42,000	1	0.503
	Distance to road	0.031	2.887	− 4.736	4.758	1.000	42,000	1	0.504
	Distance to settlement	0.009	2.896	− 4.751	4.756	1.000	42,000	1	0.501

### Temporal occurrence pattern

**Figure 3:**
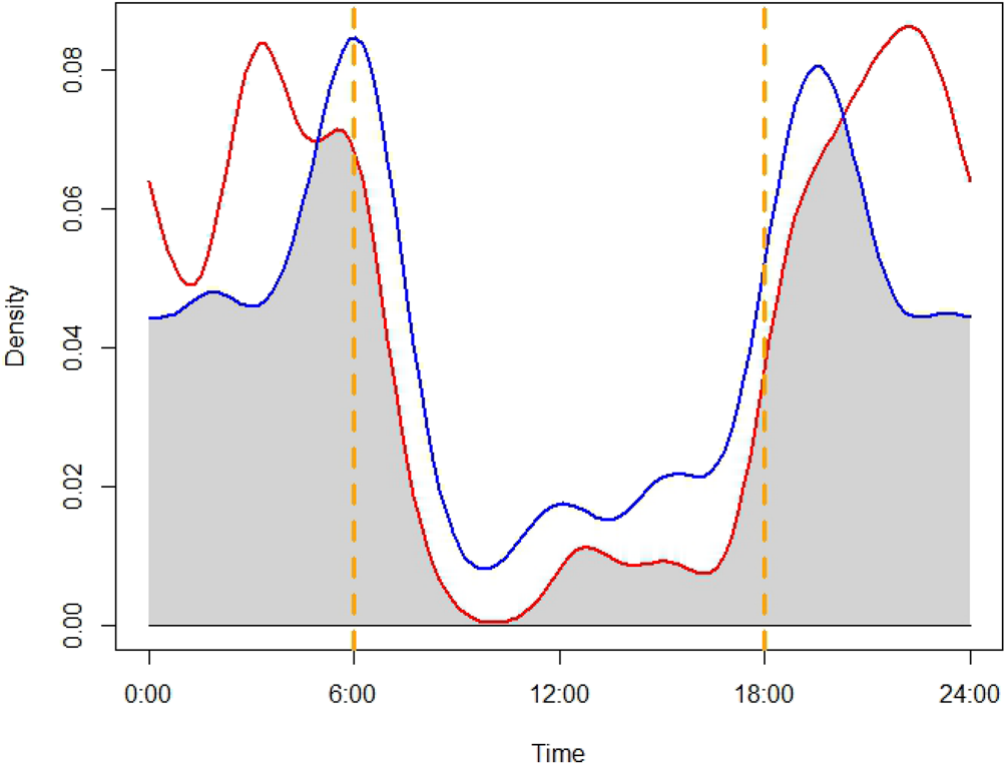
Diel overlap between leopard cats (red line) and jungle cats (blue line), Parsa–Koshi Complex, Nepal, 2022–2023. Dashed orange lines represent sunrise and sunset.

**Figure 4:**
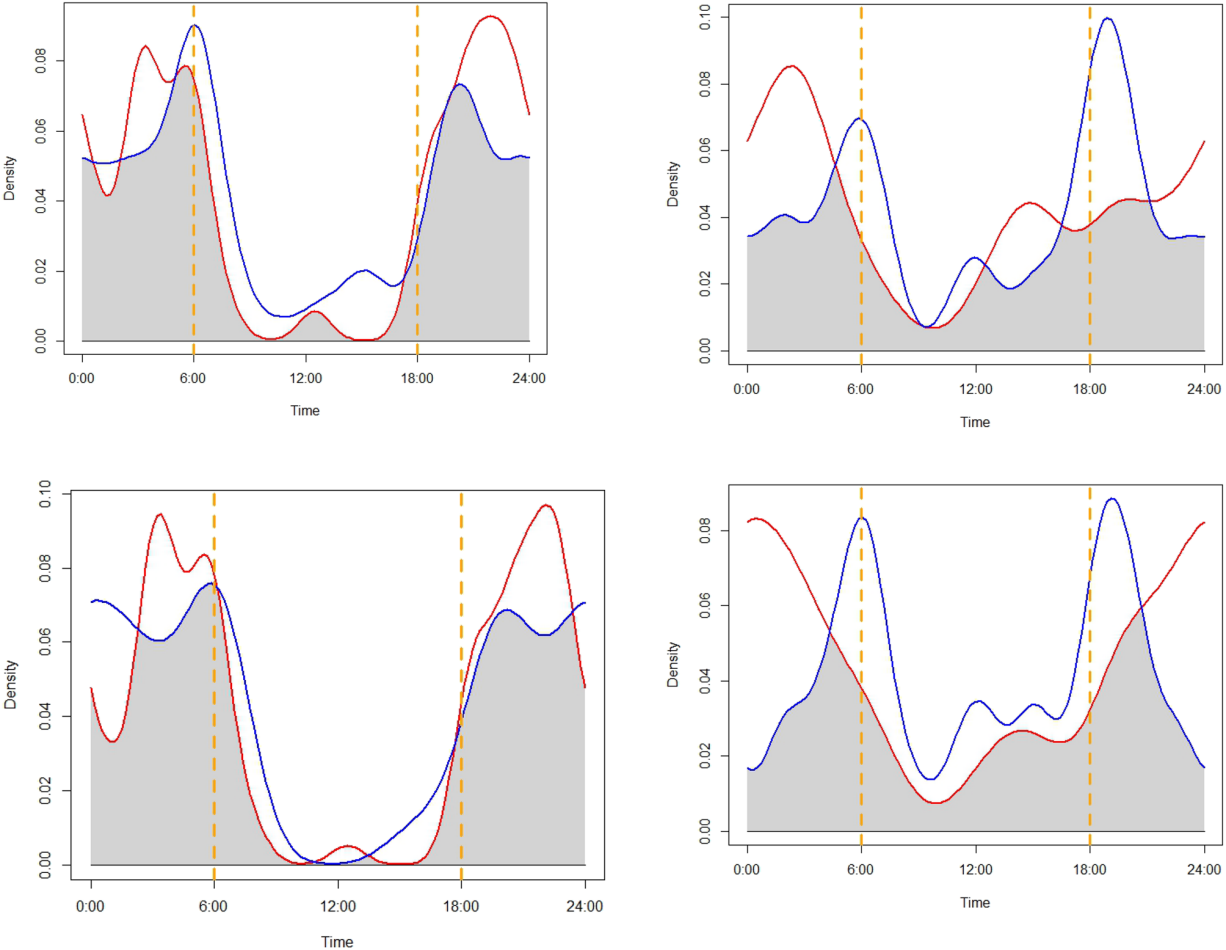
Diel detections of leopard cat (red line) and jungle cat (blue line) inside protected area (upper left), outside protected area (upper right), in presence of large predators (lower left) and in absence of large predators (lower right), Parsa–Koshi Complex, Nepal, 2022–2023. Dashed orange lines represent sunrise and sunset.

We observed high diel overlap between leopard cats and jungle cats (Dhat1 = 0.803, norm0CI: 0.724‒0.889), with both species largely nocturnal (Fig. 3). Leopard cats were mostly active during 18:00‒24:00 h, followed by 2:00‒5:00 h. Greatest jungle cat activity occurred during 4:00‒8:00 h, followed by 17:00‒20:00 h. The minimum overlap (Dhat1) of both species inside and outside protected areas were 0.803 (norm0CI = 0.711–0.902; Fig. 4) and 0.678 (norm0CI = 0.517–0.818), respectively. Overlap between the two sympatric cat species in the presence of large carnivore was 0.836 (norm0CI = 0.743–0.927), and 0.678 (norm0CI = 0.655–0.958) in absence of large predators.

## Discussion

We found that large predators, canopy cover and frequency of human detections influenced the occupancy of jungle cats or leopard cats. The presence of large predators such as tigers and leopards can influence on jungle cats and leopard cats within shared habitats^31,39^. Consequently, the presence of large carnivores can shape the behavior, habitat selection, and distribution of jungle cats and leopard cats, potentially leading to coexistence through spatial or temporal niche differentiation within the carnivore community^29–31,40^. Presence of these small cats in the same habitat as large carnivores might be due to scavenging of carcass remains from large carnivore kills^22,41^.

The jungle cat occupancy decreased with increasing canopy cover which might be due to patchy landscape, potentially resulting from habitat fragmentation and such variability in canopy cover was noticed in the PKC. Jungle cats prefer open areas, probably due to improved ability to hunt smaller rodents^24^. Further, the tufted ear tips, slender body, long limbs, short tails and cream-colored pelage of jungle cats provide better adaptive advantage in open areas^42^. Canopy cover did not influence the occupancy of leopard cats probably due to this species being a habitat generalist^20^. However, leopard cats prefer dense canopy cover, probably due to presence of small prey species such as rodents, birds, reptiles, frogs, and insects^43^. Among forests, they appear to select disturbed forests and plantations^44^. Leopard cats reportedly used forests with 75‒83% canopy cover in Singapore^43^.

The detection probability and occupancy of both cats was not influenced by nearness to water bodies, however, the proximity of water bodies may influence distributions of these species at a different spatial resolution, as they serve as important resources for jungle cats and leopard cat’s prey species for hunting near to bed reeds of riverbanks and dry streams^22^. In our study, distance to nearest settlement, major road, and number of livestock detections or detections of the other cat species did not influence occupancy of leopard cats or jungle cats. It might be due to fragmented patches of habitat between dense settlement and roads in the study area. Fragmented habitat from human settlements and roads in the study area could have caused the observed response. Habitat fragmentation can alter species movements and behavior (e.g., dispersal) through limiting available resources from widespread and abundant to localized in isolated patches (i.e. clumped resources), and mitigate the effects of nearby human activities^45^. The insignificant impact of sympatric cat species on each other could occur through spatial niche segregation or variation in habitat selection where jungle cats select more open areas^19,46^ and leopard cats forests with dense canopy^43^.

Diet activity of jungle cats and leopard cats revealed high overlap between the two species. Both cat species exhibited greater activity during early morning and evening and were largely inactive during the day. These activity patterns may be influenced by factors such as hunting behavior, prey availability, and avoidance of human disturbances. Both species are of similar size and forage on similar prey species forcing them to share similar feeding niche^22^ and being sympatric foragers, their overlap is mostly due the activity of their prey. The co-existence of these two species having overlapping diets and activity patterns suggests spatial segregation to reduce ecological overlap. The relatively low spatial overlap between species outside protected areas might correspond with habitat fragmentation in this area along with selection of jungle cats for human settlements^47^. Similarly, the high overlap between the species in areas with presence of large predators present might be due to habitat quality^48,49^ as well as providing foraging opportunities from carcasses left by them^22,41^. In addition, smaller felids can increase their activity during daily periods when top predators are less active to reduce risk^50,51^, resulting in increased activity overlap between leopard cats and jungle cats. Understanding these temporal patterns can aid in the development of conservation strategies promoting species coexistence.

## Conclusions

Our study highlights interactions and activity patterns between jungle cats and leopard cats in a fragmented landscape. Co-existence of leopard cats and jungle cats appears to occur through a combination of spatial, and temporal differentiation. We recommend future studies consider diet analyses to better understand niche differentiation between these two species. Understanding these interactions between these species, among others, is important for ensuring integrity of ecosystems and associated process along with promoting coexistence with humans.

## Materials and methods

### Study area

**Figure 5:**
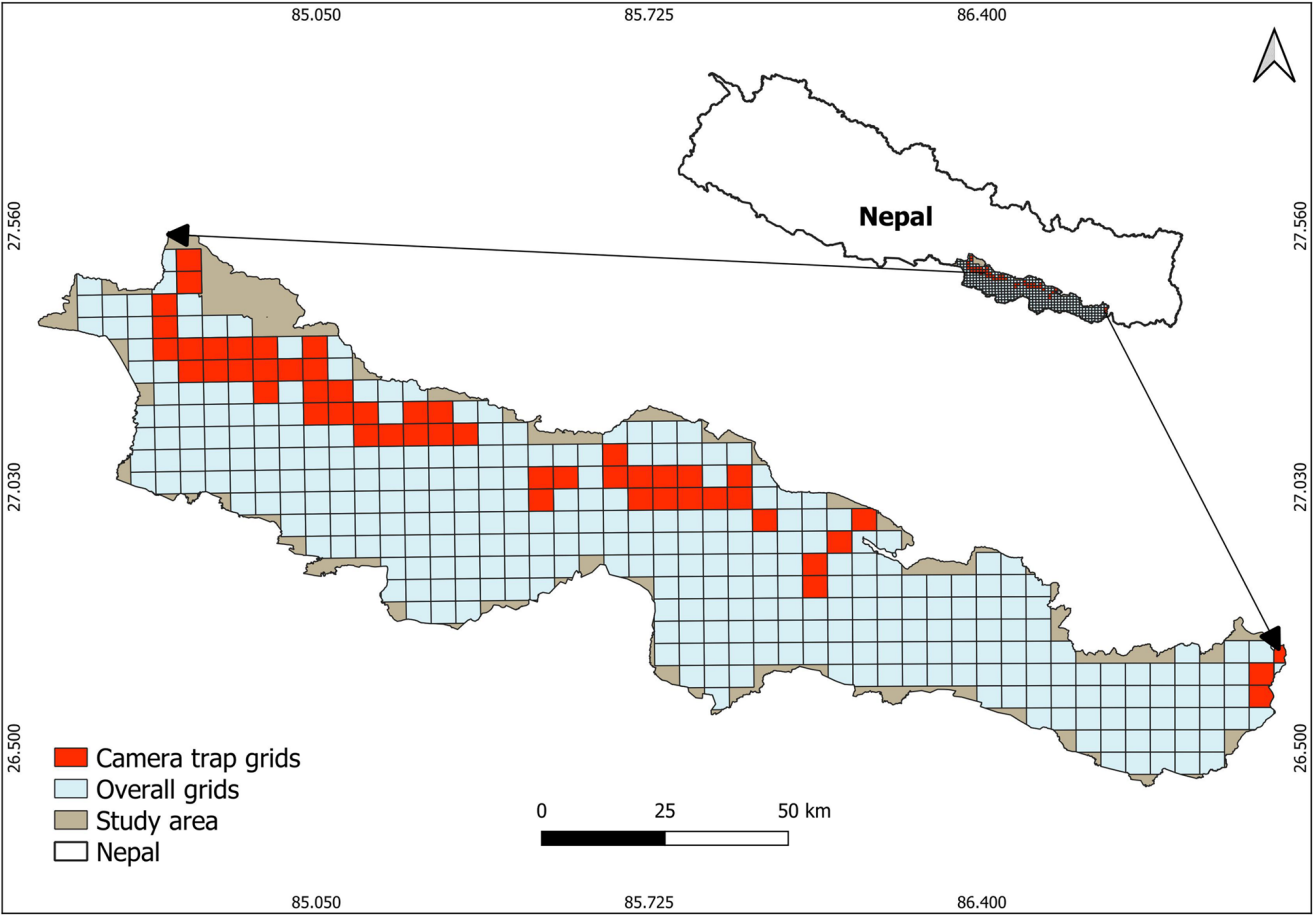
Study area for estimating jungle cat and leopard cat occupancy, Parsa–Koshi Complex, Nepal, 2022–2023. Map created in QGIS 3.24.1 of QGIS Development Team (https://qgis.org/).

We conducted this research in the PKC (9661 km^2^), Madhesh Province, Nepal, which encompasses the area between Parsa National Park (PNP) in the west and Koshi Tappu Wildlife Reserve (KTWR) in the east (Fig. 5). In addition to protected areas such as PNP and KTWR, the PKC has community-managed and national and private forests that contribute to the conservation of over 50 mammalian species and the region's rich biodiversity^20,50,52–54^. The PKC also serves as a crucial corridor for Asian elephants (*Elephas maximus*) migrating between PNP and KTWR^55^.

Elevations within PKC are 80–800 m above sea level. Major forest types are sub-tropical, including sal (*Shorea robusta*), and mixed forests dominated by acacia (*Acacia catechu*) species. Local communities residing in PKC rely on crop and livestock agriculture for their subsistence, and depend on forest products including firewood, leaves, and wood for various purposes^52,56^.

### Data collection

We collected presence data for jungle cats and leopard cats during December 2022–March 2023. We deployed 154 cameras throughout PKC maintaining a minimum distance of 1 km between adjacent cameras, excluding areas with human settlements and open farmland (Fig. 5). We left cameras in place for 3 weeks, checking each week before moving each camera to another cell. Cameras were positioned 40‒60 cm above ground along possible tracks and trails and each camera was programmed to obtain three images with a 30-s delay. The cameras were placed at random with respect to each other. We deployed cameras for 3234 trap days (154 sites × 21 days). We programmed cameras to take three images per detection with a 30-s delay between detections.

At each camera location we recorded canopy cover and distance to nearest waterbody, settlement, and major road. We extracted vector files for waterbody and major roads from OpenStreetMap^57^ and the vector layer of human settlements from the Humanitarian Data Exchange^58^. We estimated canopy cover at each camera location using the Gap Light Analysis mobile application (GLAMA;^59^. Distance to nearest settlement, waterbody, and major road was measured using using QGIS.

We identified all mammals (including humans) species from images. In addition to habitat parameters for each camera we also used number of livestock and human, and presence of large predators (tiger and leopard). Finally, we included the presence of leopard cat and jungle cat as a covariate for the other species. We standardized variables to have a mean of zero and standard deviation of 1 to account for heterogeneity^60,61^.

### Data analysis

We first performed correlation analyses for continuous variables using a threshold of |*r*| > 0.7^62^. None of the variables were highly correlated (|*r*| ≤ 0.70); thus, all were used in analyses. We used hierarchical occupancy modeling^61^ in R program^63^ to assess detection probability, naïve occupancy, and impacts of covariates on leopard cats and jungle cats. We used each week of 21 days each camera was deployed (total survey duration) as a sampling occasion representing three replicate occasions. We used two variable sets to model occupancy and detection probability. For occupancy (space use model) we used site-based factors including canopy cover percentage, large predator presence, sympatric cat species presence, number of humans detected, and number of livestock detected. For detection probability (detection model) we used distance-based factors including distance to water, distance to road, and distance to settlement. We hierarchically modeled occupancy and detection probabilities for each of the sympatric cat species. We used single species occupancy models for both species and created the object data as a matrix of species detections at each site i, where the matrix comprised the number of detections for each sampling replicate. We used occupancy as an indicator of habitat selection rather than performing spatial analysis^64–66^.

We derived occupancy as

$${\text{z}}_{{\text{i}}} \sim {\text{Bernouli}}\left( \psi \right),$$

where *z* is a latent variable that can be drawn from detection histories and *z*_*i*_ is drawn from a Bernouli distribution with the parameter probability *ψ*. We then modeled detection probability as a binomial distribution where, if *z*_*i*_*-1, p* is the probability of success, and if *z*_*i*_*-1*, the probability of success equals zero (*yi* ~ *Binomial*(*ni, pzi*)).

Using ψ as the probability of occupancy, the equation for leopard cat was

$$\begin{aligned} logit\left( {\psi_{i} } \right) & = \beta_{0} + \beta canopycover + \beta livestock + \beta predators + \beta water + \beta human + \beta road \\ & \quad + \beta settlement + \beta junglecat \\ \end{aligned}$$

and the equation for jungle cat is given as

$$\begin{aligned} logit\left( {\psi_{i} } \right) & = \beta_{0} + \beta canopycover + \beta livestock + \beta predators + \beta water + \beta human + \beta road \\ & \quad + \beta settlement + \beta leopardcat \\ \end{aligned}$$

where, *β*_0_ = *logit*(*ψ*_*0*_).

And *β* varies for each species. We incorporated correlation between detection probability, occupancy, and intercept. β_0_ is the probability of occupancy of the species at site i with a given combinations of variables^67^.

We generated model output using Markov Chain Monte Carlo (MCMC) simulation and confirmed model convergence by evaluating Rhat value, with a threshold of 1.1^68^. We ran the adaptive MCMC simulation using the jagsUI^69^ and coda^70^ packages in program R and Just Another Gibbs Sampler (JAGS;^71^) with three chains, 1000 adaptations, 1000 burn ins and 15,000 iterations. We concluded the effect to be significant if the Bayesian credible intervals did not overlap 0. All means are reported with ± 1 standard deviation.

We derived a detection probability map of jungle cat and leopard cat occurrence using Inverse Distance Weighting (IDW) interpolation in QGIS using the tool “IDW Interpolation”^72^. We derived detection probabilities for each cell as the proportion of survey replicates in which the species was detected. We compared the difference in occupancy and detection probability of the species using t-test for both species.

We calculated activity overlap coefficient (Dhat1) of jungle cat and leopard cat using the overlap package^73^ in R program. Overlap was calculated inside and outside protected areas as well as in presence and absence of large predators. Overlap ranges from 0 (no overlap) to 1 (complete overlap) and were used to calculate the extent of overlap between the respective kernel density estimates. We defined overlap as common area under the two curves by using the minimum of the two kernel estimate at each instant^74^. We used 999 bootstraps to generate 95% confidence intervals^75^. The calculated overlap was then compared using the overlapPlot function in the overlap package.

## Data Availability

Our camera trap data are at fixed location where we detected endangered species including tigers and leopards. Because of the potential for poachers to use these sites to locate target species, we cannot make our presence data publicly available. However, we can provide these data on reasonable request to the corresponding author.
